# A Novel *GUSB* Mutation in Brazilian Terriers with Severe Skeletal Abnormalities Defines the Disease as Mucopolysaccharidosis VII

**DOI:** 10.1371/journal.pone.0040281

**Published:** 2012-07-05

**Authors:** Marjo K. Hytönen, Meharji Arumilli, Anu K. Lappalainen, Heli Kallio, Marjatta Snellman, Kirsi Sainio, Hannes Lohi

**Affiliations:** 1 Department of Veterinary Biosciences, Research Programs Unit, Molecular Medicine, University of Helsinki, Helsinki, Finland; 2 The Folkhälsan Institute of Genetics, Helsinki, Finland; 3 Biochemistry and Developmental Biology, Institute of Biomedicine, University of Helsinki, Helsinki, Finland; 4 Equine and Small Animal Medicine, Faculty of Veterinary Medicine, University of Helsinki, Helsinki, Finland; Pasteur Institute of Lille, France

## Abstract

Hundreds of different human skeletal disorders have been characterized at molecular level and a growing number of resembling dysplasias with orthologous genetic defects are being reported in dogs. This study describes a novel genetic defect in the Brazilian Terrier breed causing a congenital skeletal dysplasia. Affected puppies presented severe skeletal deformities observable within the first month of life. Clinical characterization using radiographic and histological methods identified delayed ossification and spondyloepiphyseal dysplasia. Pedigree analysis suggested an autosomal recessive disorder, and we performed a genome-wide association study to map the disease locus using Illumina’s 22K SNP chip arrays in seven cases and eleven controls. A single association was observed near the centromeric end of chromosome 6 with a genome-wide significance after permutation (p_genome_  = 0.033). The affected dogs shared a 13-Mb homozygous region including over 200 genes. A targeted next-generation sequencing of the entire locus revealed a fully segregating missense mutation (c.866C>T) causing a pathogenic p.P289L change in a conserved functional domain of β-glucuronidase (GUSB). The mutation was confirmed in a population of 202 Brazilian terriers (p = 7,71×10^−29^). GUSB defects cause mucopolysaccharidosis VII (MPS VII) in several species and define the skeletal syndrome in Brazilian Terriers. Our results provide new information about the correlation of the *GUSB* genotype to phenotype and establish a novel canine model for MPS VII. Currently, MPS VII lacks an efficient treatment and this model could be utilized for the development and validation of therapeutic methods for better treatment of MPS VII patients. Finally, since almost one third of the Brazilian terrier population carries the mutation, breeders will benefit from a genetic test to eradicate the detrimental disease from the breed.

## Introduction

Genetic disorders with significant involvement of the skeletal system form a group of clinically and genetically heterogeneous diseases arising through abnormalities in the development, growth or maintenance of skeletal structures. Over 450 skeletal conditions have been recognized in human with variable etiologies, challenging the diagnosis of the diseases [1]. Most of the skeletal disorders are monogenic and can be classified based on their clinical, radiographic, molecular and biochemical features.

The main component of bone and other connective tissues is extracellular matrix (ECM), which mainly consists of collagen and proteoglycans. ECM has an essential role in skeletal development which is proven by numerous amounts of skeletal disorders arising through mutations in the genes encoding the structural proteins like aggrecan, perlecan or several types of collagens causing collagenopathies and osteogenesis imperfecta (OI) [1]. Skeletal deformities are also a prominent feature in certain lysosomal storage diseases (LSD) called mucopolysaccharidoses (MPS), in which deficiencies of lysosomal enzymes involved in the degradation of glycosaminoglycan (GAG) chains of the proteoglycans cause an accumulation of GAGs in lysosomes of many different cell types, mainly in the connective tissue. Eleven different forms (MPS I to IIIA,B,C and IV to IX) have been characterized in human and categorized based on the underlying enzyme deficiency [2]. Although clinical characteristics differ between mucopolysaccharidoses and even within a particular MPS type, they also share features such as dwarfism, undeveloped epiphyseal centers, dysostosis multiplex, facial dysmorphia, corneal clouding and organomegaly [2].

Dog is a unique species with extreme morphological variation. Particular skeletal features have been intentionally selected in many breeds and they have become fixed breed characteristics. For example, the length of the legs varies massively across breeds. Short legs caused by chondrodysplasia are a distinguishable feature in many breeds such as Dachshund or Basset Hound and associate with an extra functional copy of *Fgf4* gene [3]. Abnormal growth of craniofacial bones leads to brachycephaly in Boxers, Bulldogs and other short muzzled breeds. This trait was recently mapped to chromosome 1 [4]. Besides extensive and benign morphological variation among breeds, dogs have detrimental skeletal disorders. For example, various forms of OI have been characterized and linked to mutations in the *COL1A1*, *COL1A2* and *SERPINH1* genes [5]–[7].

Annotation of the canine genome and subsequent development of genomic tools provides a unique approach to tackle the genetics of skeletal disorders in a large animal model with a genetically isolated population structure. Canine skeletal disorders resemble human conditions and it is not surprising that most of the known genes have been orthologues of the corresponding human syndromes [3], [5]–[8]. As a part of our ongoing canine genomics projects, we have encountered Brazilian Terriers with severe skeletal defects. Brazilian Terrier is a small smooth-coated native Brazilian breed with a history descending from crosses of local Brazilian small dogs with imported European terriers. The breed is popular in its native country but almost unknown elsewhere except in Finland, which has one of the largest populations outside Brazil with ∼150 annual registrations within the last five years.

We aimed here to describe the clinical and pathological features of the skeletal disease in Brazilian Terriers and find its genetic cause. Using a genome-wide association study we mapped the disease to a ∼13-Mb region of *Canis lupus familiaris* chromosome 6 (CFA6) and subsequent targeted next-generation sequencing (NGS) identified a novel missense mutation in the affected puppies in the *β-glucuronidase* (*GUSB*) gene, which causes MPS VII. Our study establishes a new spontaneous canine model for human MPS VII and enables the development of a genetic test to eradicate this detrimental syndrome from the breed.

## Results

### Clinical Features Indicate Ambulatory Dand Dwarfism

**Figure 1:**
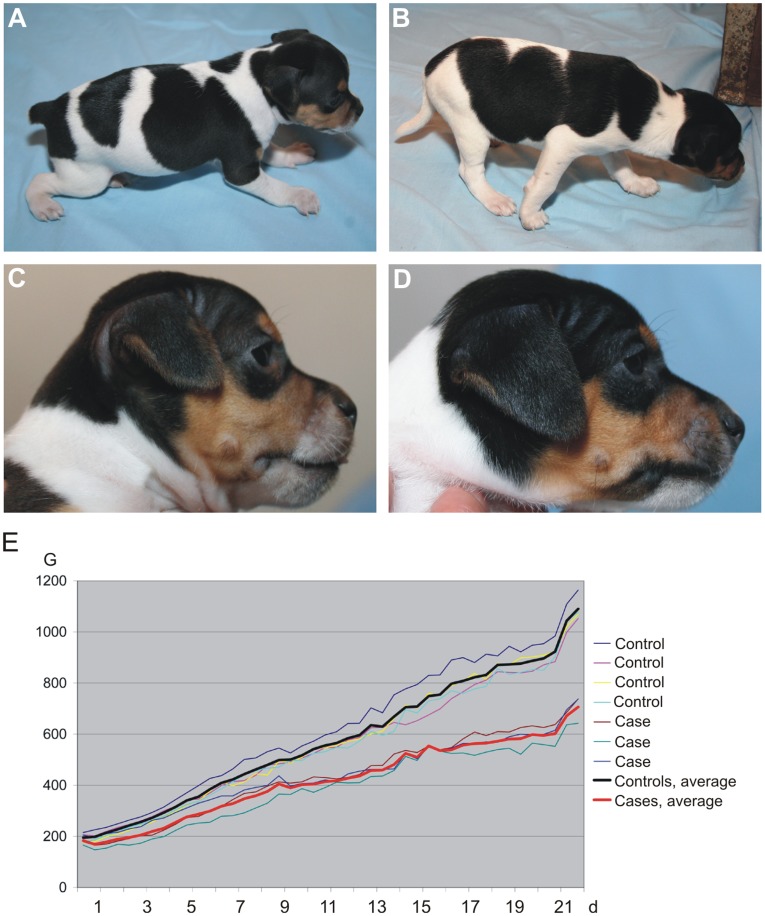
Morphological features of the affected Brazilian Terriers. Deformed legs with crooked radiocarpal joints and prominent joint hyperlaxity especially in the hind limbs (A) and a brachycephalic craniofacial morphology (C) presented by a 4-week-old Brazilian Terrier puppy compared to a healthy littermate (B,D). The affected puppies (n = 3) were severely growth retarded and weighted 35% less than their healthy littermates (n = 4) at the age of three weeks (E).

A litter of seven Brazilian Terriers was presented to clinic because three of the puppies showed multiple severe skeletal defects at the age of 4 weeks. General clinical examination and radiography was performed for the affected puppies and their gender-matched healthy littermates and the healthy dam to explore the cause for skeletal deformities. The affected puppies showed normal behavioural activities and appetite but they were unable to walk and function properly (**Video S1**). The congenital and progressive signs became evident within the first four weeks of life. All affected puppies presented similar phenotypic characteristics with brachycephalic craniofacial morphology, dwarfism and deformed legs with crooked radiocarpal joints and prominent joint hyperlaxity especially in the hind limbs (**Fig. 1A,C**). Ophthalmoscopic examination revealed no pathological changes in the eyes. The weights of the three affected puppies and four normal littermates were measured at birth and followed until 3 weeks of age. The affected puppies showed severe growth retardation and had 35% less weight than their healthy littermates at the age of three weeks (**Fig. 1E**). Later, several other related Brazilian Terrier litters became available and altogether 15 affected dogs from 8 different litters were confirmed to have similar clinical signs, though, the severity of the phenotype varied even among the affected littermates. Due to poor prognosis the affected puppies were euthanized between 2 to 6 weeks of age except the two that died spontaneously at the age of 1 and 3 weeks for unknown reasons. Both sexes were equally affected.

### Radiological Findings Reveal Spondyloepiphyseal Dysplasia

**Figure 2:**
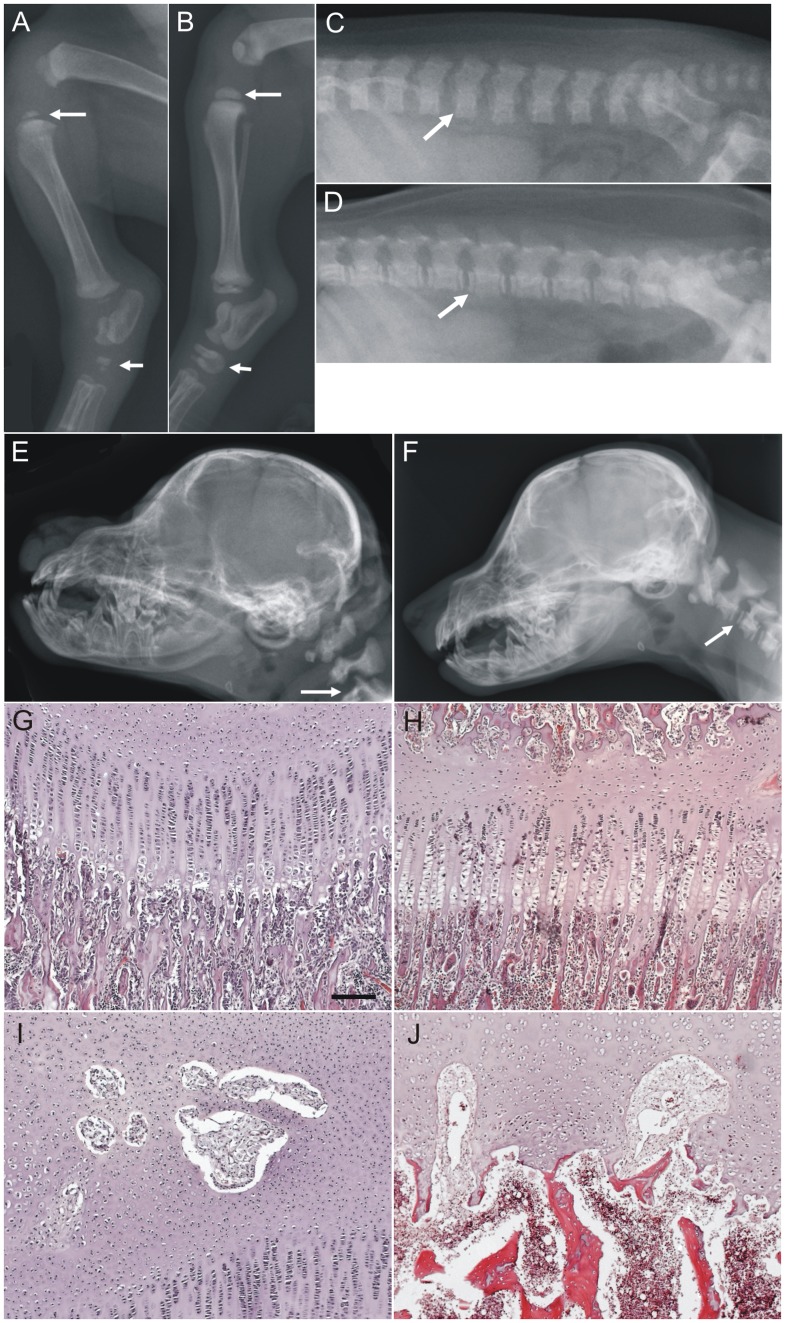
Radiographic and histological features of skeletal disease in Brazilian Terrier puppies. Comparison of the hind limbs of an affected 4-week-old puppy (A) and a healthy littermate (B) revealed epiphyseal dysplasia in the affected dog. The ossification centers of the epiphyses of the proximal tibia (long arrow) and tarsal bones (short arrow) are smaller in the affected puppy while the epiphyses of the healthy littermate are normally mineralized. The vertebral endplates of the lumbar spine (arrow) are poorly mineralized in the affected (C) puppy as compared with the healthy littermate (D). The skull of an affected 6-week-old Brazilian Terrier (E) has brachycephalic appearance with shortened maxilla. Radiopacity of the bones is reduced. The cervical vertebral endplates are not mineralized (arrow). A skull of a normal littermate is shown in comparison (F). Histological examination of the skeletal structures of a 4-week-old affected puppy showed normal growth plate with resting, proliferating and hypertrophic layers of chondrocytes in the tibia (G) but lack of secondary ossification center and occasional separate spots of loose fibrous tissue without sign of osteogenesis (I). The lack of secondary ossification centers and irregularities of the growth plate were present in the vertebral bodies (J). The epiphysis of a 5-week old healthy puppy is shown in comparison (H). Scale bar 100 µm.

Seven affected dogs and three age- and sex-matched controls were radiographed to investigate the structural abnormalities. In addition, radiographs were taken from the dam of the first litter. Epiphyseal dysplasia of long bones and vertebral endplates with delayed ossification of the epiphyseal cartilages was evident in all affected dogs (**Fig. 2A,C,E**). Additionally, the maxilla was short (prognathia inferior), the calvarium was widened, and the ossification centers of the cuboid bones of carpus and tarsus were reduced in size when compared to the normal littermates (**Fig. 2A-D**). In the affected dogs skeletal radiopacity was generally slightly decreased, but the cortices of the long bones appeared normal. All affected individuals had also marked carpal and tarsal joint effusion (**Fig. 2A**). The healthy littermates and the dam had no skeletal changes.

### Histopathological Findings Indicate Delayed Ossification

Postmortem tissues were collected from eight euthanized puppies for histopathological analyses. During the post-mortem necropsy, all the puppies were noted to have domed skulls and thin and opaque skull bones. The long bones were soft and cortical bone was thin. The carpal and tarsal bones were cartilaginous and soft. The ligaments were soft and there was joint hyperlaxity. Histological examination of the skeletal structures of the affected puppies showed all structures of the growth plate, resting, proliferating and hypertrophic layers of chondrocytes, in the long bones (**Fig. 2G**). The secondary ossification centers in the epiphyses were either small or completely lacking and occasionally there were several separate spots of loose fibrous tissue without any sign of osteogenesis (**Fig. 2I**). The carpal and tarsal bones were composed mainly from cartilage. The ossification centers were either lacking or small and included thin and irregular primary trabeculae. The vertebral bodies had occasionally irregular cartilage columns in the growth plate and inclusions of loose fibrous tissue (**Fig. 2J**). No gross abnormalities in the other tissues were found.

### GWAS Maps the Disease to CFA6

**Figure 3:**
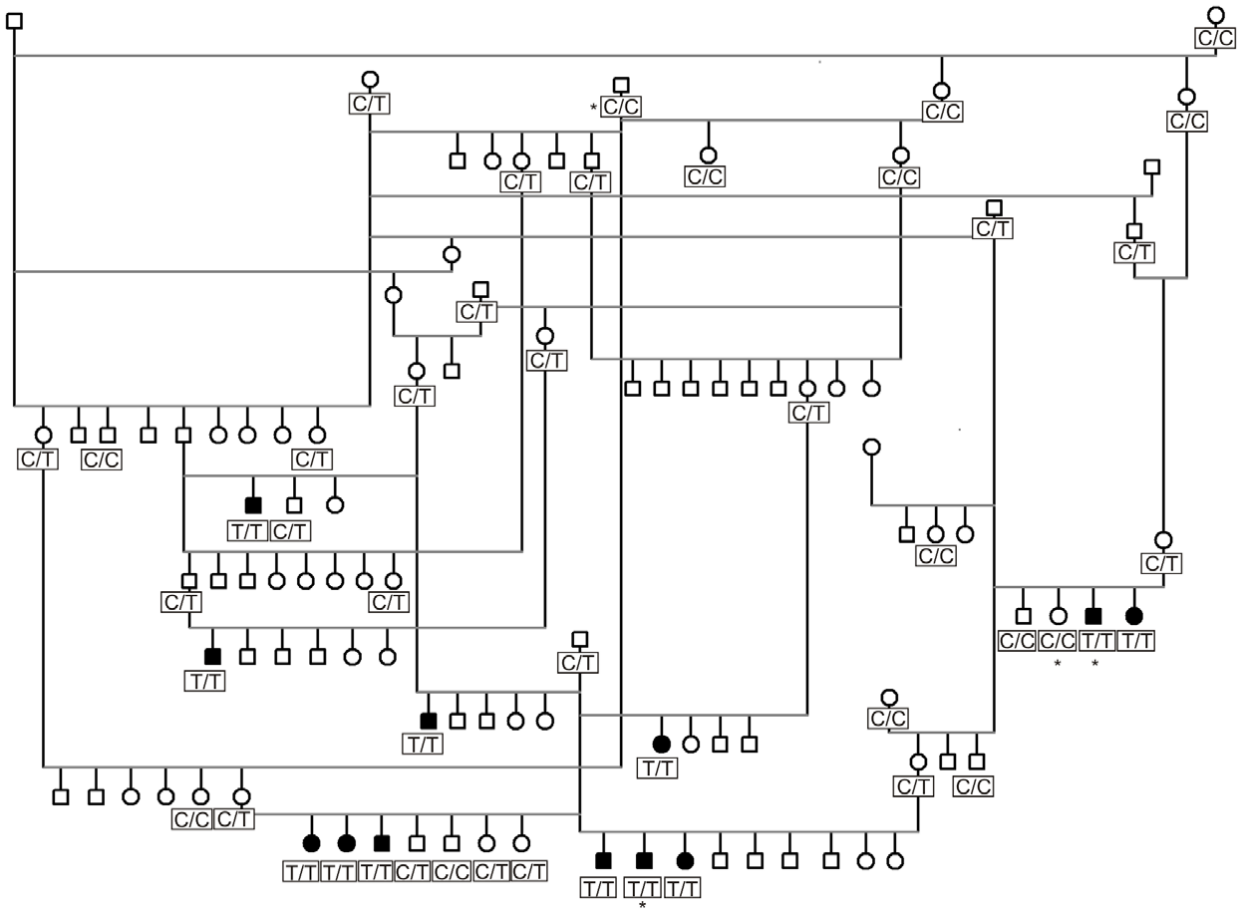
An example of our large Brazilian Terrier pedigree established around the affected puppies. The pedigree with multiple affected dogs in the litters, equal gender distribution and healthy parents suggests a recessive mode of inheritance. Squares note males and circles females. Black-filling indicates affected dogs. Genotypes of the *GUSB* gene (T/T = homozygous mutation allele, C/C = homozygous wild type allele) are shown below the boxes. Dogs used in the NGS are marked by asterisks.

A large pedigree tracing all the affected dogs back to a common Brazilian ancestor was established (**Fig. 3**). Pedigree analysis suggested an autosomal recessive mode of inheritance. There were several affected puppies in each litter and all parents were normal. Both males (n = 7) and females (n = 5) were affected. The proportion of the affected dogs in the litters was 31,6%, slightly over an average of 25% assumed for a recessive condition.

**Figure 4:**
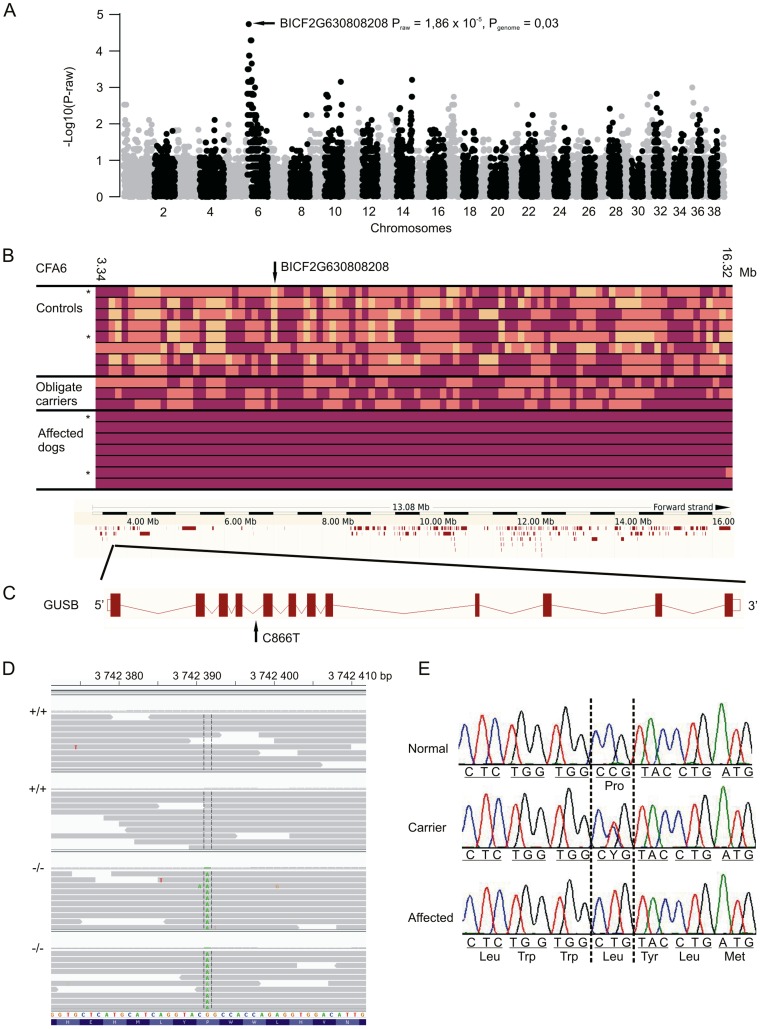
A genome-wide association study reveals an associated locus at CFA6. Manhattan plot indicates the best SNP with a raw and an empirical p-value after 100 000 permutations (A). Affected dogs share a 13-Mb homozygous haplotype, which contains over 200 genes (B). Colors in haplotype block are as follows: plum marks the homozygous allele, orange heterozygous allele and yellow an opposite homozygous allele. Dogs used for the NGS are marked by asterisks. A targeted sequencing of the entire locus identified a homozygous c.866C>T mutation in the exon 5 of the *GUSB* gene (C). The mutation was found by next generation sequencing (D) and was validated by Sanger sequencing (E).

We performed a genome wide association study (GWAS) to map the disease locus with Illumina’s 22K canine SNP chips in a cohort of 18 dogs including 7 cases and 11 controls. A case-control association test revealed significant association on CFA6 with the best SNP (BICF2G630808208) at 7,265, 846 Mb (p_raw_ = 1,86×10^−5^, p_genome_ = 0,03313) (**Fig. 4A**). Assessment of genotypes at the CFA6 locus revealed a long shared homozygosity block in the affected dogs spanning from 3, 341,099 bp to 16, 324,133 bp (**Fig. 4B**). In addition, all three obligate carriers (parents of the affected dogs) were heterozygous for the haplotype. The region is in the vicinity of the centromeric end of the CFA6 challenging the exact definition of the beginning of the homozygous block. Homozygous block contained 137 SNPs of which none showed a complete segregation between cases obligate carriers and other controls (**Fig. 4B**).

### Next Generation Sequencing Identifies a Missense Mutation in GUSB

The associated homozygous region contains at least 210 annotated or predicted genes (**Fig. 4B**) including several genes with possible role in the bone formation. We selected two candidate genes for further investigation, namely *Procollagen C-endopeptidase* *enhancer* (*PCOLCE*) and *SMAD ubiquitin regulatory factor 1* (*Smurf1*). PCOLCE binds specifically to the type I procollagen C propeptide and has a role in collagen maturation [9], a process in which defects have been linked to several skeletal disorders. Smurf1 has an essential role in the regulation of osteoblast differentiation [10], [11]. We sequenced all exons, except the first exon of *Smurf1* gene, in both genes including exon-intron boundaries and splice sites and 5′- and 3′-UTRs from two affected puppies and two obligate disease carriers. We did not find any segregating coding variant in either of the genes.

Due to a large number of genes in the genomic region we decided to proceed with a next generation sequencing approach. The entire 13-Mb associated region was captured from two cases and two controls with opposite haplotypes (**Fig. 4B**) using NimbleGen’s in-solution capture method followed by a paired-end NGS by HiSeq2000. We found altogether ∼13000-19000 SNPs and ∼5000 indels in each dog (**Table 1**). The variant lists between cases and controls were then compared to discover possible disease-causing variants. Besides the two Brazilian Terrier controls we used 32 additional healthy dogs from four different breeds as controls to filter out common variants. We found two novel homozygous exonic SNPs, one deletion and one insertion unique for cases. The 9-bp deletion was located in *monoacylglycerol O-acyltransferase 3* (*MOGAT3*) gene (at position BROADD2:6:11681043) which is an intestinal specific enzyme implicated in fat absorption [12]. The 3-bp insertion was located in *olfactory receptor, family 2, subfamily AE, member 1* (*OR2AE1*) gene (at position BROADD2:6:12694743). Neither of these genes were potential candidate genes for the studied disease due to their known restricted function outside the skeletal system. One of the exonic SNPs was synonymous and the other non-synonymous. The non-synonymous exonic variant was found in the exon 5 (c.866C>T) of the *β-glucuronidase* (*GUSB*) gene, resulting in a proline to leucine substitution at amino acid position 289 (p.P289L).

**Table 1:** SNP and indel statistics of the NGS of the identified locus in Brazilian Terriers.

	Sample	No of reads	%mapped	Coverage	Total no of variants		No of variants in coding regions	
					SNPs	Indels	SNPs	Indels
**Control**	1	6303701	99.90	48	20555	6214	578	28
	2	6776456	99.92	52	19530	6240	640	23
**Case**	3	6834323	99.86	52	13044	4781	549	27
	4	7060250	99.91	54	12742	4505	558	28
**Case-specific**	**All variants**				410	154	10	3
	**Novel homozygous variants**				291	52	2	2

The 13,2-Mb locus at CFA6 was captured and sequenced in two cases and two controls with opposite haplotypes. Variants were discovered by comparing the target sequence with the reference sequence. Ensembl’s CanFam2.0 was used as a reference. The variants present only in the affected dogs were found by comparing the two Brazilian Terriers cases with the two breed controls and 32 other dogs from four breeds without any skeletal diseases.

The GUSB enzyme is a lysosomal homotetramer [13]. The human and canine monomers consist of 651 amino acids and human protein includes three functional domains comprised of residues 22–223, 224–342 and 343–632. The canine GUSB is 81% similar to human GUSB at amino acid level [8]. The first domain of the GUSB monomer forms a distorted barrel-like structure with a jelly roll motif and two β-hairpin loops. The second domain is similar to the immunoglobulin constant domains and the third forms the active enzyme site with α/β- or TIM-barrel motif [14]. The identified p.P289L substitution of GUSB in the affected Brazilian Terriers resides in the middle of the second domain. A multiple alignment of this affected domain across eukaryotic and prokaryotic species revealed that the substitution site and its surrounding residues are well conserved (**Fig. 5**). The disruption of the conserved region by the substitution is likely to impair the GUSB structure and function, which was further supported by a PolyPhen-2 software predicting a “probably damaging” effect of the mutation with a maximum score 1.0.

**Figure legend:**
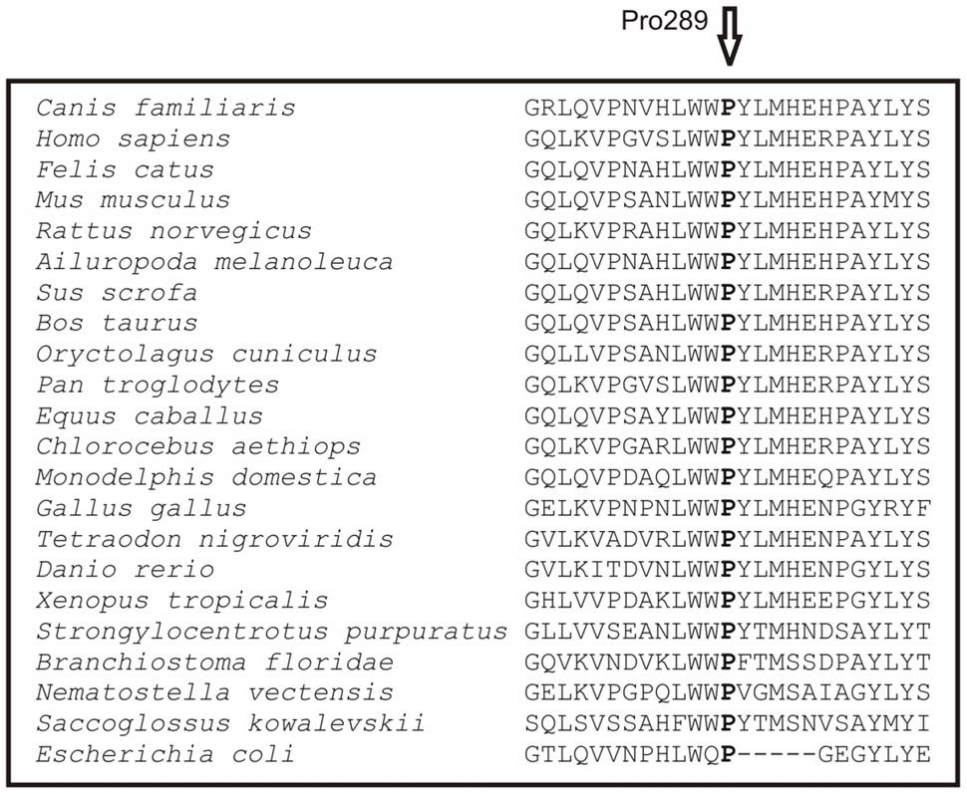
The conservation of the *GUSB* mutation site. A multiple alignment of the substitution site (P289) indicates a high conservation across taxa including eukaryotic and prokaryotic species. The p.P289L mutation is predicted to be pathogenic and likely disrupts the structure of the GUSB enzyme.

For the validation of the c.866C>T variant as a causative mutation segregating with the disease we genotyped a set of 202 Brazilian Terriers available from our canine DNA bank including 15 cases, 12 obligatory carriers and 175 healthy controls. Among the 187 healthy dogs, 134 dogs were homozygous for the wild type allele and 53 dogs were heterozygous for the c.866C>T variant. All affected dogs were homozygous and all obligatory carriers heterozygous for the variant. The results confirmed a significant association of the c.866C>T variant with the disease (p = 7,71×10^−29^). The carrier frequency among the healthy population was 28,3%. We sequenced an additional panel of 166 dogs from 36 different breeds to further strengthen the causality of the mutation. None of the additional dogs carried the mutation. Collectively, these results validate the disease locus identified through GWAS and indicate the full segregation of the mutation with the recessive disease.

### The Affected Dogs have Elevated Urinary GAGs and Substantially Decreased β-glucuronidase Activity

Deficient β-glucuronidase activity leads to an elevation of GAGs in urine and therefore the quantitative measurement of urinary GAGs are commonly used in screening patients for mucopolysaccharidosis. We performed a colorimetric quantification of urinary GAGs from three affected puppies (∼6 weeks of age) and three healthy age-and breed-matched controls to investigate the functional effect of the mutation. All the affected dogs were genotyped homozygous for the mutation and controls for wild type allele. The total urinary GAGs were measured biochemically in relation to creatinine levels. We found an average of 2.7-fold increase in GAGs in the affected dogs (**Table S2**). In addition, we determined the activity of β-glucuronidase in the serum of the three affected dogs and six control dogs from which three were heterozygous for the mutation (carrier) and three were homozygous for the wild type allele (normal). In the affected dogs, the enzyme activity was severely impaired with a mean residual activity of 0,2% compared with the wild type dogs (**Table 2**). The GUSB activity in the carrier dogs was also reduced to 67% further supporting the detrimental effect of the mutation for the GUSB function. The activity of another lysosomal enzyme, α-mannosidase, was within the control range, although much more variability was observed in the study cohort. GUSB has been shown to be stable in the serum upon storage while α-mannosidase and several other lysosomal enzymes are more unstable which might explain the more wide range in the measured activity [15]. These results confirm the predicted pathogenic effect of the GUSB mutation and demonstrate the disease in Brazilian Terriers to be MPS VII.

**Table 2:** Lysosomal enzyme activities in the serum of affected and healthy Brazilian Terriers.

	Range (*n*)		
Enzyme	Affected	Carrier	Normal
β-glucuronidase	0,05–0,31 (3)	72,4–102,6 (3)	85,7–151,1 (3)
α-mannosidase	802,7–833,1 (3)	1190,7–2078 (3)	560,6–2624,9 (3)

The activity of β-glucuronidase and α-mannosidase was measured from the serum samples of three affected, carrier and normal dogs. Activity is expressed as µmol/l/h.

## Discussion

This study characterizes a severe developmental disorder in Brazilian Terriers and identifies its genetic cause. The affected dogs suffer from multiple skeletal defects, growth retardation and they have failure to thrive leading to a premature death or euthanasia within the first weeks of life. Pathological and radiological studies revealed a prominent spondyloepiphyseal dysplasia due to a delayed ossification. The genetic cause of the recessive disease was mapped to CFA6 resulting in the identification of the causative mutation in the *GUSB* gene. The *GUSB* mutation defines this canine skeletal syndrome as a mucopolysaccharidosis VII, a lysosomal storage disease affecting mainly connective tissue.

The *GUSB* gene encodes a lysosomal β-glucuronidase that degrades GAGs, including heparan sulfate, dermatan sulfate, and chondroitin-4,6-sulfate. Deficient β-glucuronidase activity causes MPS VII (Sly syndrome) [16]. *GUSB* mutations have been described in many species including human, mouse, cat and dog. Typical features in human patients (OMIM #253220) include mental retardation, skeletal deformities, corneal clouding and hepatosplenomegaly. The clinical variability among human is extensive ranging from a prenatal lethality to mild skeletal abnormalities with normal intelligence. Almost 50 unique mutations have been described in human. The site of the mutation in the *GUSB* gene correlates with the residual enzymatic activity and related clinical severity [17]. Only a small percentage of normal GUSB activity (2–3%) can protect against a severe phenotype.

Besides human, seven spontaneous or induced murine MPS VII models have been reported [18]–[22]. All models present similar clinical, morphological and histopathological characteristics but the severity of the clinical signs depends on the strain. The phenotypical features in mice are comparable with human MPS VII patients including shortened life span, dysmorphic facial features, skeletal dysplasia and widespread lysosomal storage of GAGs in various tissues.

Feline GUSB deficiency has been described in three separate cases but the causative mutation has been identified only in one of them [23]–[25]. All feline models share clinical similarities including a young age of onset, dwarfism, facial dysmorphism, walking difficulties, corneal clouding, epiphyseal dysplasia of the vertebra and long bones, and vacuoles in several tissues. The causative mutation, p.E351K, in one population of cats affects a highly conserved residue [25].

Canine MPS VII have been previously identified in two cases, a mixed breed dog and a German Shepherd [26], [27]. The first clinical signs in these dogs appeared at 2 to 5 months of age and involved weakness of the hind legs followed by a progressive dysfunction of all limbs. The affected dogs presented also other typical MPS VII features including growth retardation, facial and other skeletal dysmorphisms, and corneal clouding. Joints were extremely lax and easily subluxated and radiographic examination showed severe epiphyseal dysplasia. Abnormalities in several other organs were also present including hepatomegaly, tracheal dysplasia and cardiac abnormalities. MPS VII was caused in both cases by the p.R166H substitution resulting in a severe reduction of the GUSB enzymatic activity [8], [27].

We described a novel canine *GUSB* mutation, p.P289L, in Brazilian Terriers with a more severe skeletal phenotype. The affected puppies had a prominent limb dysfunction and they were never able to ambulate properly. However, unlike the previous canine models, Brazilian Terriers did not have corneal clouding or detectable abnormalities in other organs. This could be explained by the young age (∼4 weeks) of the affected dogs at the time of euthanasia. For example, corneal opacity became evident in the other canine models at 8 weeks of age and in cats at few months of age [24]–[26]. As it is known that the gradual accumulation of undegraded GAGs in the lysosomes correlates with the progressive nature of the disease, in most human and animal models the clinical signs appear gradually. Regardless of the differences, most features in Brazilian Terriers were similar to the other canine, feline, murine and human MPS VII, including growth retardation, craniofacial dysmorphisms, excessive joint laxity and epiphyseal dysplasia with the involvement of the long bones and vertebral end plates. The earlier onset of the disease in Brazilian Terriers compared to the other canine and feline models suggests that the mutation is highly detrimental for the GUSB enzymatic activity. The p.P289L mutation affects a conserved site of the second functional immunoglobulin-like domain in GUSB and thus, likely alters GUSB structure or function. Indeed, the determination of the β-glucuronidase activity in the serum of affected puppies showed that the residual activity was near absent (0,2%) and was also reduced in the carrier dogs confirming the pathogenic nature of the p.P289L mutation. Defective enzymatic activity of GUSB leads to elevated secretion of GAGs into urine and the affected puppies in this study had threefold elevation in urinary GAGs. Our clinical, genetic and biochemical data confirms the disease as MPS VII.

In summary, we describe a severe canine skeletal disease and identify a syndrome-defining mutation in the *GUSB* gene. This study provides new information about genotype-phenotype correlation in a very early form of the disease and establishes a novel canine model for MSP VII. In general, there are two primary treatment options, enzyme replacement therapy (ERT) or hematopoietic stem cell transplantation (HSCT) that can provide substantial benefits in MPSs in human patients [28]. For example, MPS I (Hurler disease) patients are successfully treated by both ERT and HSCT [29]–[33]. Currently, there is not an efficient treatment for MPS VII. Gene therapy has been successfully performed in dogs using retroviral vectors expressing canine GUSB [34]. Our canine patients could be utilized for the development and validation of such therapies, which could eventually be advantageous for both dogs and human. Meanwhile, we have developed a genetic test to eradicate the disease from the breed, in which almost every third dog carries the mutation.

## Materials and Methods

### Study Cohort

The research group was contacted by breeders about an unknown debilitating skeletal dysplasia in Brazilian Terrier puppies. To investigate the clinical and genetic characteristics of the disease a breed-wide collection was initiated to sample both affected and control dogs. EDTA-blood samples were collected altogether from 202 privately owned Brazilian Terriers including 15 affected puppies and 187 healthy controls. The affected animals came from eight different litters with the average age of the onset of the clinical signs being 3 weeks. A large pedigree was established around the affected dogs using the GenoPro genealogy software (http://www.genopro.com/) and utilizing the public dog registry by the Finnish Kennel Club (http://jalostus.kennelliitto.fi) to evaluate the mode of inheritance (**Fig. 3**). Blood samples were stored at −20°C until genomic DNA was extracted using the Puregene DNA Purification Kit (Gentra Systems). DNA concentration was determined with the ND-1000 UV/Vis Spectrophotometer (NanoDrop Technologies). Collection of blood samples was approved by the Animal Ethics Committee at the State Provincial Office of Southern Finland (ESLH-2009-07827/Ym-23).

### Radiological and Histological Studies

Seven affected puppies, a dam and three healthy littermates from five different litters were radiographically examined to study the structural abnormalities. A laterolateral radiograph of the whole body and laterolateral as well as ventrodorsal radiographs of the skull were obtained. Affected and healthy puppies were photographed and video recorded. Ophthalmoscopic examination was performed for the three affected puppies by a board-certified veterinary ophthalmologist. General post mortem examination was performed for three affected puppies from the same litter. Autopsies were taken from different tissues including lung, kidney, spleen, heart, pituitary gland, brain, eye, long and short bones of the limbs and spine. We performed histological analyses from tissue samples (limb bones, spine, skull and mandible with teeth) for four additional affected puppies to further explore the microscopic changes during osteogenesis. We used a Brazilian Terrier puppy without the skeletal disease but that had died due to intestinal infection at 5 weeks of age as a control dog in the histochemical analyses. Tissue samples were fixed in 10% formalin for 48 hours, decalcified in Morse’s solution or in 10% EDTA, dehydrated and embedded in paraffin. Paraffin blocks were cut in to 5µm sections and stained with hematoxylin and eosin.

### Genome Wide Association Study

Seven affected dogs from four nuclear families and eleven control dogs (**Fig. 3**) were genotyped using Illumina’s CanineSNP20 BeadChip of 22,362 validated SNPs. Healthy control dogs included three obligate disease carriers (parents), two non-affected siblings and six other related healthy dogs. The genotype data was filtered with a SNP call rate of >95%, array call rate of >95% and minor allele frequency of >5%. Based on these criteria 366 SNPs were removed for low genotyping efficiency and 5647 SNPs for low minor allele frequency. No individual dogs were removed for low genotyping and no SNPs were removed because of significant deviations from the Hardy-Weinberg equilibrium (p< = 0.0001). After frequency and genotyping pruning, 16595 SNPs remained for analyses. Basic case-control association test was performed by PLINK [35]. Genome-wide significance was ascertained with phenotype permutation testing (*n* = 100,000).

### Mutation Screening and SNP Validation

Mutation screening of exons of *Smurf1* and *PCOLCE* genes including exon-intron junctions and 5′- and 3′ -UTRs was performed using two clinically and patholocigally confirmed cases and two obligate disease carriers. Primers were designed for exon 5 of the *GUSB* gene to validate the c.866C>T variant and PCR was performed for 202 Brazilian Terriers including 15 affected dogs, 12 obligatory carriers and 175 additional healthy dogs. In addition, a panel of 166 dogs from 36 different breeds was genotyped for the variant (Table S1). Primer sequences are available upon request. PCR reactions were carried out in a total reaction volume of 20 µl with 20 ng of genomic DNA using a standard PCR protocol. PCR products were purified with ExoSAP-IT (GE Healthcare) and sequenced with a 3730×l DNA Analyzer (Applied Biosystems) and analyzed using Variant Reporter v1.0 (Applied Biosystems).

Web-based software PolyPhen-2 (genetics.bwh.harvard.edu/pph2) [36] was applied to evaluate the pathogenic effect of the mutation. The score range for PolyPhen-2 is from 0 to 1 with the threshold “probably damaging” at 0.85.

### Target Enrichment and Next Generation Sequencing

We performed a targeted sequence capture and next generation sequencing to identify the disease-causing mutation. We used NimbleGen’s in-solution capture technology to enrich a 13,2-Mb target region (CFA6: 3,250,000-16,400,000) for sequencing (Roche NimbleGen). Probes for the target region were designed by Roche NibleGen (Roche NimbleGen, Madison, WI, USA). We selected two Brazilian Terrier cases and controls with opposite haplotypes for target enrichment and sequencing. The same targeting experiment contained also samples from our other targeting projects including 8 Border Terriers, 12 Duck Tolling Retrievers, 8 Schipperkes and 4 White West Highland Terriers and these samples were used as additional controls.

Three micrograms of genomic DNA was sheared using Covaris-S2 focused sonicator using following (duty cycle 10%, intensity 5 and 200 cycles per burst). Subsequent end repair, A-tailing and ligation were performed using NEBNext® DNA Sample Prep Master Mix Set 1 (New England BioLabs, Ipswich, MA, USA) ) according to manufacturer’s protocol with following modifications: (1) 1µl of the 25µM Illumina Index PE adapter oligo mix (from primers 5′-GATCGGAAGAGCACACGTCT-3′ and 5′-ACACTCTTTCCCTACACGACGCTCTTCCGATCT-3′) was used in adapter ligation. (2) After each consecutive library preparation step the libraries were purified with Agencourt AMPure XP -beads (Beckman Coulter, Brea, CA, USA). The volume of the beads was 1.8 times the volume of the reaction. (3) 15 ng of each ligated library was used for pre-capture PCR amplification in 5 parallel reactions per library with 14 amplification cycles. (4) The initialization step in the amplification was 2 minutes instead of 30 seconds and the denaturation step was 20 seconds instead of 30 seconds. (5) Illumina’s three primer multiplexing scheme was replaced by a direct two primer amplification using universal forward primer (AATGATACGGCGACCACCGAGATCTACACTCTTTCCCTACACGACGCTCTTCCGATCT) and an index primer (CAAGCAGAAGACGGCATACGAGATXXXXXXGTGACTGGAGTTCAGACGTGTGCTCTTCCGATCT). The latter oligonucleotide contained a sample specific 6 base pair index tag where XXXXXX is presented. All primers and oligonucleotides were custom ordered from Sigma Aldrich (St. Louis, MO, USA).

The solution based enrichment was performed according to the manufacturer’s instructions with following modifications: hybridization enhancing oligonucleotides (PE-HE1 and PE-HE2) were replaced with 5′-CAAGCAGAAGACGGCATACGAGAT-3′, 5′-GTGACTGGAGTTCAGACGTGTGCTCTTCCGATCT-3′ and 5′- AATGATACGGCGACCACCGAGATCTACACTCTTTCCCTACACGACGCTCTTCCGATCT-3′to enhance performance on indexed libraries. Prepared sample libraries were sequenced using Illumina’s HiSeq 2000 sequencing platform according to manufacturer’s instructions (PE 101 bp reads). Sample preparation, target enrichment experiments and variant calling pipeline analysis [37] was performed by the core facility, Institute for Molecular Medicine Finland (FIMM, Technology Centre, University of Helsinki, Helsinki, Finland). Canine genome build CanFam2 was used as reference sequence. Further data analysis was performed using open source R language and environment (http://www.r-project.org) and ANNOVAR software for annotation of the genetic variants [38].

### Biochemical Studies

Urine samples were collected from three affected and three healthy Brazilian Terrier puppies. Urinary GAG levels were measured using a protocol for colorimetric quantification of GAGs based on de Jong et al. [39]. The final values were expressed as GAG/creatinine ratios. Serum samples were collected from three affected and six control Brazilian Terriers including three heterozygous carriers and three wild type dogs. The samples were stored and shipped at −20°C to the laboratory of Oulu University Hospital where the activity of β-glucuronidase and α-mannosidase was determined according to the routine protocols.

## Supporting Information

Video S1 **Ambulatory defects presented by a MPS VII-affected Brazilian Terrier puppy at 4 weeks of age.**
(WMV)

Table S1 **Summary of the** ***GUSB*** **genotypes at nucleotide position 866 in 41 different breeds.** The genotyping was performed by Sanger sequencing for all the samples except 12 Nova Scotia Duck Tolling Retrievers, 4 West Highland White Terriers, 8 Border Terriers and 8 Schipperkes for which the genotypes were collected from the NGS data. Genotypes are denoted as C/C = homozygous wild type allele, C/T = heterozygous carrier, T/T = homozygous mutation allele.
(DOC)

Table S2 **Urinary GAG analysis in the affected Brazilian Terriers and the healthy controls.** The urinary GAG/creatinine ratios for the three affected Brazilian Terrier puppies indicate elevated excretion of GAGs in the urine as compared to the controls from same breed and age.
(DOC)
