# Supplementary material for: A Novel GUSB Mutation in Brazilian Terriers with Severe Skeletal Abnormalities Defines the Disease as Mucopolysaccharidosis VII
Source: PLoS One. 2012 Jul 5;7(7):e40281. doi: 10.1371/journal.pone.0040281 (PMC3395332; doi:10.1371/journal.pone.0040281)
Supplement: Table S1 — Summary of the GUSB genotypes at nucleotide position 866 in 41 different breeds. The genotyping was performed by Sanger sequencing for all the samples except 12 Nova Scotia Duck Tolling Retrievers, 4 West Highland White Terriers, 8 Border Terriers and 8 Schipperkes for which the genotypes were collected from the NGS data. Genotypes are denoted as C/C = homozygous wild type allele, C/T = heterozygous carrier, T/T = homozygous mutation allele. (DOC) [file pone.0040281.s002.doc]

**Table S1. Summary of the *GUSB* genotypes at nucleotide position 866 in 41 different breeds.**

|  | **Number of dogs** | | | |
| --- | --- | --- | --- | --- |
| **Breed** | **Total** | **C/C** | **C/T** | **T/T** |
| Airedale Terrier | 4 | 4 | 0 | 0 |
| American Cocker Spaniel | 4 | 4 | 0 | 0 |
| Australian Cattle Dog | 4 | 4 | 0 | 0 |
| Australian Silky Terrier | 4 | 4 | 0 | 0 |
| Australian Terrier | 4 | 4 | 0 | 0 |
| Bedlington Terrier | 4 | 4 | 0 | 0 |
| Bichon Frisé | 8 | 8 | 0 | 0 |
| Border Terrier | 8 | 8 | 0 | 0 |
| Boston Terrier | 4 | 4 | 0 | 0 |
| Brazilian Terrier | 202 | 134 | 53 | 15 |
| Brittany | 4 | 4 | 0 | 0 |
| Bull Terrier | 4 | 4 | 0 | 0 |
| Cairn Terrier | 3 | 3 | 0 | 0 |
| Chihuahua | 12 | 12 | 0 | 0 |
| English Cocker Spaniel | 4 | 4 | 0 | 0 |
| English Toy Terrier | 8 | 8 | 0 | 0 |
| French Bulldog | 2 | 2 | 0 | 0 |
| German Pinscher | 7 | 7 | 0 | 0 |
| Havanese | 4 | 4 | 0 | 0 |
| Italian Greyhound | 4 | 4 | 0 | 0 |
| Jack Russell Terrier | 8 | 8 | 0 | 0 |
| German Hunting Terrier | 4 | 4 | 0 | 0 |
| Miniature Pinscher | 8 | 8 | 0 | 0 |
| Miniature Schnauzer | 4 | 4 | 0 | 0 |
| Norfolk Terrier | 2 | 2 | 0 | 0 |
| Nova Scotia Duck Tolling Retriever | 12 | 12 | 0 | 0 |
| Papillon | 2 | 2 | 0 | 0 |
| Parson Russell Terrier | 10 | 10 | 0 | 0 |
| Phalène | 2 | 2 | 0 | 0 |
| Poodle | 5 | 5 | 0 | 0 |
| Portuguese Podengo | 2 | 2 | 0 | 0 |
| Pug | 4 | 4 | 0 | 0 |
| Pyrenean Shepherd | 4 | 4 | 0 | 0 |
| Schipperke | 8 | 8 | 0 | 0 |
| Soft-Coated Wheaten Terrier | 4 | 4 | 0 | 0 |
| Spanish Water Dog | 4 | 4 | 0 | 0 |
| Staffordshire Bull Terrier | 4 | 4 | 0 | 0 |
| Standard Schnauzer | 4 | 4 | 0 | 0 |
| Tibetan Terrier | 4 | 4 | 0 | 0 |
| West Highland White Terrier | 7 | 7 | 0 | 0 |
