# Supplementary material for: A Novel GUSB Mutation in Brazilian Terriers with Severe Skeletal Abnormalities Defines the Disease as Mucopolysaccharidosis VII
Source: PLoS One. 2012 Jul 5;7(7):e40281. doi: 10.1371/journal.pone.0040281 (PMC3395332; doi:10.1371/journal.pone.0040281)
Supplement: Table S2 — Urinary GAG analysis in the affected Brazilian Terriers and the healthy controls. The urinary GAG/creatinine ratios for the three affected Brazilian Terrier puppies indicate elevated excretion of GAGs in the urine as compared to the controls from same breed and age. (DOC) [file pone.0040281.s003.doc]

**Table S2. Urinary GAG analysis in affected Brazilian Terriers and healthy controls.**

| **Sample** | **GAG/crea** |
| --- | --- |
| Affected 1 | 69,0 |
| Affected 2 | 88,2 |
| Affected 3 | 84,6 |
| Control 1 | 32,4 |
| Control 2 | 28,8 |
| Control 3 | 27,3 |
